# The challenges for women’s health in sub-Saharan Africa: Lessons learned from an integrative multistakeholder workshop in Gabon

**DOI:** 10.7189/jogh.11.02002

**Published:** 2021-09-04

**Authors:** Marrium Habib, Ayola Akim Adegnika, Josiane Honkpehedji, Stefanie J Klug, Silvia Lobmaier, Kathrin Vogg, Amaya L Bustinduy, Andreas Ullrich, Jutta Reinhard-Rupp, Meral Esen, Clarissa Prazeres da Costa

**Affiliations:** 1 Institute for Medical Microbiology, Immunology and Hygiene, Technical University of Munich (TUM), Munich, Germany; 2 Center for Global Health, TUM School of Medicine, Technical University of Munich (TUM), Munich, Germany; 3 Institute for Tropical Medicine (ITM), University Clinic Tübingen, (UKT), Tübingen, Germany; 4 Centre de Réchèrches Médicales de Lambaréné (CERMEL), Lambaréné, Gabon; 5 Chair of Epidemiology, Department of Sport and Health Sciences, Technical University of Munich (TUM), Munich, Germany; 6 Clinic and Polyclinic for Gynecology, University Hospital, Klinikum Rechts der Isar (MRI), Technical University Munich (TUM), Munich, Germany; 7 Department of Clinical Research, London School of Hygiene and Tropical Medicine (LSHTM), London, United Kingdom; 8 Charité – Universitätsmedizin Berlin, Germany; 9 Ares Trading S.A., Eysins, Switzerland, an affiliate of Merck; 10 German Center for Infection Research (DZIF), Tübingen, Germany

The sub-Saharan African (SSA) region is home to more than 230 million females of reproductive age who face multiple intersecting health, social, gender and economic challenges [1]. Neglected tropical diseases (NTDs) are a group of chronic disabling, almost exclusively communicable diseases affecting the poorest of the poor, especially in Africa, which alone bears about 40% of the global burden of NTDs [2-4]. While both men and women are impacted, biological and sociocultural biases mean that NTDs disproportionately affect women and girls [5]. In recent decades, there has been a global shift from communicable toward non-communicable diseases (NCDs), which cause almost 32 million deaths in low- and lower-middle-income countries (LMIC) [6]. It is expected that by 2030, 85% of NCD-related deaths among women will occur in LMICs, including many countries of SSA region [7]. For women older than 50, NCDs are the leading cause of both death and disability-adjusted life years (DALYs) [8]. Important disparities persist in access to maternal and reproductive health services both within and between countries in SSA [9]; it is estimated that almost half of the women in SSA do not have access to essential health care during pregnancy and childbirth. In 2017, SSA accounted for roughly two-thirds of all maternal deaths in the world [10]. Hence, it is evident that many, if not most, women and girls in SSA carry a triple burden of vulnerability to NTDs, NCDs and poor reproductive health outcomes. Here, we report on the outcomes of an integrative, multistakeholder workshop held in Gabon, Central Africa, to help develop a framework for synergistic, sustainable and gender- and context-appropriate interventions to manage the NTD-NCD complex and additionally reproductive health.

## Rationale for tracer conditions

This workshop identified female genital schistosomiasis (FGS), cesarean section (CS) and cervical cancer (CC) as tracer clinical conditions for the key themes of NTDs, NCDs and reproductive health respectively because they constitute major women’s health challenges in the SSA that demand collaborative solutions. Here we would like to highlight that while integrative solutions for the NTD-NCD complex have been attempted, we go one-step further to integrate reproductive health into our framework. The underlying reasoning was based on epidemiological data and gaps in knowledge outlined in brief as follows:

### Female genital schistosomiasis (FGS)

Human schistosomiasis (bilharzia) is a parasitic disease prevalent in tropical areas with well-known clinical urinary an gastrointestinal tract manifestations. Female genital schistosomiasis (FGS) is a neglected and disabling disease that results when eggs from the waterborne parasite *Schistosoma haematobium* are trapped in the human reproductive tract. Clinical manifestations include hypertrophic and ulcerative lesions of the vulva, vagina, and cervix, as well as grainy sandy patches, abnormal blood vessels, and rubbery papules on the cervix or vaginal wall. Infected females may suffer from symptoms mimicking sexually transmitted infections (STIs). Fresh water contact has been identified as an especial risk factor, hence, rural communities that rely on agriculture and fishing are especially at risk [11]. Socio-cultural factors in SSA increase the risk for developing urogenital schistosomiasis and thus FGS in women and girls eg, they are 2/3rds more likely than men to carry out water-based domestic and/or recreational activities [12]. The anti-helminthic drug Praziquantel (PZQ), is cheap, easily available, and effective in treating early infections [13]. The WHO recommends annual treatment with PZQ as a relatively affordable intervention, in highly endemic areas to treat schistosomiasis. Persistence of human papilloma virus (HPV), the causative agent for CC, has also been associated with FGS [14]. FGS has also been associated with horizontal transmission of HIV, rendering it a major cofactor in the AIDS epidemic [15]. FGS does not find a mention in most medical textbooks, or even in the lay media compounding low awareness and poor diagnosis of the condition, among health workers [16]. Diagnosis usually requires facilities for colposcopy and histopathology, which are not widely available in endemic areas. In specialized gynecological or obstetric clinics, characteristic FGS lesions may not be attended to because of the lack of knowledge about the disease manifestations. In fact, health care providers have been reported to confuse the symptoms of FGS with those of STI or CC [17]. Out-of-pocket (OOP) cost for obtaining PZQ outside of school-based deworming and mass drug administration (MDA) programs is often prohibitive and results in low uptake by women [18]. Thus, obtaining accurate disease burden information, especially community-level burden assessment, is daunting, given that the clinical awareness is low, and diagnosis is complex and cumbersome. Moreover, treatment is often inaccessible. Incorrect diagnosis and management can have profound psychosocial implications for sexually active girls. The effect of the disease on fertility and pregnancy have been reported to cause marginalization, stigma, isolation and the threat of gender-based violence (GBV) for women [19].

### Cervical cancer (CC)

Research conducted over the past 30 years has established that HPV is the primary cause of cervical cancer. The majority of women become infected within a few years after becoming sexually active with HPV types 16 and 18 causing about 70% of cases worldwide [20,21]. Infection rates for women tend to be high during their teens and 20s and about 10% of infected women develop precancerous lesions. If not detected through screening programs (and then treated), precancer develops into invasive cancer [22]. The lack of effective screening and treatment programs make CC a tracer disease of inequity and the inability to access adequate health care; about 90% of CC-related deaths occurring among women in LMICs and most in SSA [22]. The inequitable proportion of this health burden is expected to worsen, as global mortality rates in low-income countries will be nearly 27%, compared with only 1% in high-income countries [23]. HPV infection could effectively be prevented by vaccination. However, infrastructural weaknesses of the health systems in SSA prevent many girls and women from receiving the vaccine [24]. In fact, only six (Botswana, Lesotho, Rwanda, Senegal, South Africa and Uganda) out of 46 SSA countries have introduced national HPV immunization programs in any form [25,26]. A study of four West African countries showed that less than 1% of women have ever been screened [27]. In Gabon, CC ranks first in mortality for cancer in women and accounted for 18% of all new cancers diagnosed in Gabonese women in 2018 [28]. Additionally, evidence suggests that HIV-HPV coinfection might lead to a more rapid transition from intra-epithelial lesions of the cervix to invasive cancer [29-31]. The WHO‘s Global Strategy to Accelerate the Elimination of Cervical Cancer, launched in November 2020, outlines three key steps: vaccination, screening and treatment. Successful implementation of all three could reduce more than 40% of new cases of the disease and 5 million related deaths by 2050. While officially an NCD, the global outlook on CC is similar to that on NTDs: it is a disease of poverty and neglect, affecting poorer populations; has a disproportionately negative impact on female morbidity with enhanced stigma and discrimination; and can be managed and possibly eliminated through effective, feasible low-cost solutions [32].

### Cesarean section (CS)

In 2017, SSA accounted for approximately 66% of the estimated global maternal deaths [33]. One evidence-based intervention for reducing maternal morbidity and mortality is promoting institutional deliveries, attended by a skilled birth attendant (SBA) and access to CS [34]. CS is a surgical procedure widely performed to save maternal and fetal lives, preventing complications during labour and an important indicator of accessibility to emergency obstetric care [34]. The WHO considers CS rates of 10-15% to be the optimal range for this life saving interventions for mother and infant [35]; in countries where at least 10% of women have CS, the number of preventable maternal and newborn deaths show a significant decrease [36]. The African region has the lowest CS rate in the world and access to safe CS is estimated at only 1–2% in SSA [37,38]. Various studies from LMICs have identified a range of factors that contribute to sites where skilled care is available; women's lower social status, education levels, lack of autonomy in decision-making and cultural norms that discourage the use of facility-based care [39-43, 44]. A poor formal referral system for identifying high-risk pregnancies has been recognized and established a need to target women earlier for professional intrapartum care [43,44]. A Medicins sans Frontiers (MSF) multi-country analysis conducted in SSA, reported that the most common indications for CS were obstructed labor, malpresentation, history of prior CS, fetal distress, uterine rupture, and antepartum hemorrhage [45]. Multiple studies conducted across Tanzania, Ethiopia and Burkina Faso found that suboptimal management occurred in many cases of CS and that there was a lack of awareness of evidence-based guidelines, leading to unnecessary CS [46-50]. Global CS rates are rising, but inconsistently so among and within countries of the SSA and mounting evidence suggests that CS may be utilized predominantly by wealthy urbanites [51-54]. While higher rates suggest improper selection of CS, lower rates suggest an unmet need. Thus, there is an overarching concern that access to and capacity of SSA to provide CS to those women for whom it is indicated, in all settings, is lacking.

## Rationale for integrated, life course approach

**Figure 1:**
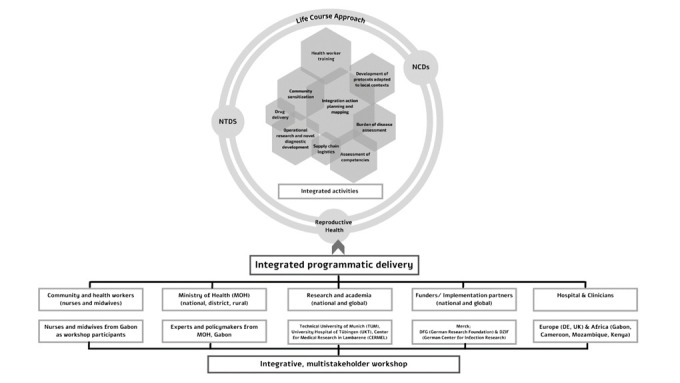
Rationale for integrated, life-course approach for multistakeholder workshop.

There is growing evidence that NTDs endemic in SSA have chronic long-term complications that can develop into NCDs [55,56]. Disability arises due to delayed diagnosis and treatment, and/or because of disease complications that are not well managed. Therefore, affected persons may end up with permanent impairments, including reduced intellectual development, which in turn may lead to functional limitations, barriers to social participation and reduced quality of life, social stigma and exclusion and chronic mental health problems [57,58]. Together, these problems incur an enormous economic and social cost to individuals, families and societies. There is also compelling evidence to suggest that many of the most prominent NTDs have sufficient diagnostic tools and treatment currently available to eliminate them as severe public health problems [59]. Historically, ministries of health in disease-endemic countries have supported management through independent, often parallel, programs and integrating multiple interventions is considered costly [60]. Within SSA, the investment in management of morbidity and disability of all three conditions continues to lag behind due to various factors: inadequately equipped health facilities, high human resource absenteeism rates, limited disease surveillance along with scarce laboratory diagnostic capacities, and insufficient government investment [61] which limit multi-sectoral and cross-sectoral collaborations and present challenges to a synergistic approach. In our approach, we attempt to build upon existing NTD-NCD intervention frameworks to integrate reproductive health. The life course approach is an intuitive method to conceptualize integrated prevention and control for NCDs and NTDs by providing a comprehensive and sustainable framework to identify key interventions for improved health literacy and knowledge translation and a systems thinking approach [62]. An integrated approach also supports social justice and equity by provision of services that are coherent, uniform, and high quality, and enhances the motivation, skill, and competence of health care workers. This is reflected graphically in **Figure 1****.**

## METHODS

An integrative, multistakeholder workshop was organized in Gabon as an explorative method to investigate how such an approach across the three key themes (with the three tracer clinical conditions) could be mastered. More specifically, we aimed to:

- Explore the level of knowledge among health care providers and assess the care systems in place for combating the local burden of NTDs like FGS and NCDs like CC, with knowledge-sharing and practical teaching modules
- Exchange comparative experiences and good practices for CS in the Gabonese context, with knowledge-sharing and practical teaching modules
- Identify opportunities for operational research collaboration between Europe and Gabon to enhance a systems-thinking approach for women’s health

The workshop was designed and developed by the HelmVit consortium (funded by the DFG, with additional assistance from the DZIF) to explore the effects of Schistosomiasis-associated pregnancy on vitamin D metabolism. The coordination team was sited at the Technical University of Munich (TUM), Germany in close collaboration with partners at the University of Tübingen (UKT), Germany and Centre de Recherches Médicales de Lambaréné (CERMEL), Gabon. An equitable representation of experts from from Europe (Germany, Switzerland and United Kingdom) and Africa (Gabon, Mozambique, Kenya and Cameroon) presented over the course of two-and-a half days. A detailed list of presenters is given in Appendix S1 in the **Online Supplementary Document**. Presentations given by international experts included an overview of the global and local picture in Gabon about the three key themes through their tracer disease-conditions, the current available epidemiological data and disease burden and the gaps in knowledge and research. Four sessions of in-depth plenaries covered the three key themes by lectures and open dialogue among participants via round tables and panel discussions. A general ‘women’s health’ plenary aimed to integrate expertise across the academic, community, industry and policy and governance spectrum. Two practical teaching modules allowed hands-on training for midwives, nurses and gynaecologists, and introduced innovative digital health applications for field diagnosis and awareness generation. A further collaborative session allowed all participants to generate reflections for knowledge exchange. A detailed agenda is reproduced in Appendix S2 in the **Online Supplementary Document**. The workshop was run in English to facilitate the full compendium of participants and researchers from diverse language backgrounds and contexts. However, since the operational language of the nurses and midwives was French, English-to-French translation facilitators were on hand for support. All English presentations were pre-translated into French and distributed as handouts to all participants. During the round-table sessions, in which participants discussed competencies, both French and English were supported options. The coordination team recorded all sessions in documentary and video format.

## RESULTS

### Outcome of interactive sessions

**Figure 2:**
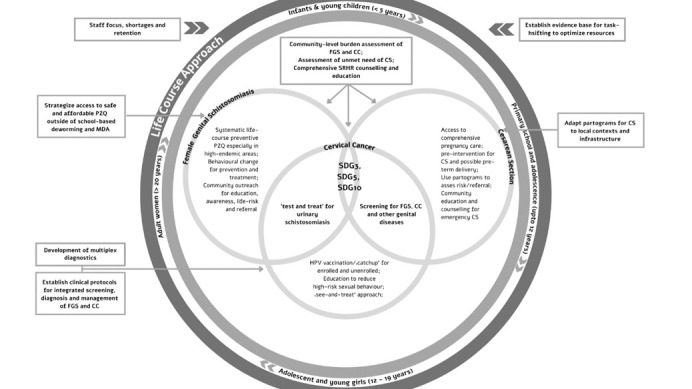
Conceptual and interventional framework for an integrated approach for management of FGS, CS and CC.

The interactive sessions instigated cross-sectoral and interdisciplinary communication to achieve three distinct outcomes: (1) To understand barriers and enablers for diagnosis, management and care of NCDs, NTDs and reproductive health conditions in the context of Gabon; (2) To instigate stakeholder engagement to explore potential mutual leverage within the interests and capacities of stakeholders; (3) To develop an integrative framework for sustainable, gender and context appropriate interventions, including identifying gaps in research (see below). We have summarized a conceptual and interventional framework for an integrated approach to the three key themes in **Figure 2****.**

### Knowledge sharing and capacity building via practical modules

The workshop also introduced hands-on training module on various modes of vaginal delivery using birth models; and novel point-of-care (POC) screening and diagnostic schemes piloted in Zambia: a portable colposcope with image analysis via integrated mobile app and possibility to integrate CC/FGS diagnosis via diagnosis from remote specialists. The content of all training modules is presented in **Table 1****.**

**Table 1:** Training content for practical modules

	FGS Cervical Cancer (CC)	Cesarean Section (CS)
	− Innovations in digital health for diagnostics	
**Practical Module**	− Portable colposcope with image analysis	− Hands-on training session with delivery model eg, management of shoulder dystocia, vaginal vacuum extraction and breech presentation delivery
	− Hands-on training session with models	
**Knowledge Sharing**	− Videos: Cervical self-swabbing, challenges in the field, learnings from Zambia BILHIV* study	- Videos: Performance of ‘normal’ CS using Misgav-Ladach method and ‘difficult’ CS with reverse breech extraction
	− Introduction of integrated cervical cancer/FGS diagnostics via m-health	- Video demonstration for birth maneuvers

FGS – female genital schistosomiasis

*The BILHIV study is a cooperation between LSHTM, University of Zambia and collaborators from Leiden Medical Center, Oslo University, the Natural History Museum and The Liverpool School of Tropical Medicine and aims to explore the innovative role of self-swabs for the diagnosis of female genital schistosomiasis.

### Operational research inventory

The operational research needs highlighted during the workshop included: (i) community-level burden assessment of FGS and CC in Gabon (ii) strategies to improve community engagement with FGS to facilitate behavioural change (iii) clinical treatment protocols and integrated management for FGS and CC – programmatic alignment within the Pink October (‘Octobre Rose’) (A public health campaign by the Sylvia Bongo Foundation, Gabon aimed at strengthening awareness-raising actions, prevention and detection for female cancers) campaign in Gabon (iv) identify best strategies to ensure access to safe, affordable and accessible PZQ for women and girls outside of school-based deworming and MDA (v) adaptation of partograms for CS to local contexts and infrastructure (vi) establish evidence base for the success of task-shifting to optimize resources eg, midwives and nurses to implement HPV vaccination and FGS-CC screening programs. These operational research needs are envisioned as contributing to an ‘Inventory of Knowledge Gaps and Research Questions for Women’s Health in SSA’ as a living document. As the life-course approach establishes, women’s health needs change during the various stages of their lives hence there is a continuous need for age and sex disaggregated data to monitor women’s health status across age categories.

### The future of integrated programmatic approaches in the context of COVID-19

The COVID-19 pandemic represents one of the most challenging global public health emergencies in almost a century. The repurposing of health care staff for the pandemic response has created challenges for the NTD, NCD and reproductive health programs in many countries [63,64]. Hence, while the gains made before the COVID-19 pandemic can cushion recurrence and rebound for many tracer disease-conditions, the impact of the pandemic could be devastating in the long term, if not addressed appropriately. Integration of the COVID-19 response, and indeed further emergencies, into existing control and elimination strategies for the three key themes is the need of the hour. As a first step, the capacity of integrated NTD-NCD-reproductive health programs could be leveraged to convey outbreak-related messages and interventions in hard-to-reach populations [65]. Existing health care infrastructure could be used for mapping preventive chemotherapy diseases and delivering drugs for both NTDs and outbreaks of novel diseases [66,67]. Alternative strategies to support high-risk groups with existing NCDs and provision of selected sexual and reproductive health and rights (SRHR) services through telemedicine (advice by telephone or online means) to replace in-person consultations have already reported some success [64]; SMS-based systems can be used for parallel reporting of COVID-19 testing along with diagnostics and/or and follow-up of NTDs, NCDs and reproductive health services in endemic regions [68]. The scaling-back of non-COVID-19-related health and social services in SSA meant the major brunt fell on women as the default family caregivers and the majority of front-line health workers, especially nurses and midwives [69,70]. Women's socially prescribed care roles typically place them in a prime position to identify trends in the ebb and flow of an outbreak; thus incorporating women's voices and knowledge could be empowering and improve emergency preparedness and response. The momentum on control and management of COVID-19 should be leveraged to inform the development of integrated programmatic approaches. This will allow us to raise the profile of the disease-conditions that affect the world’s most poor and marginalized while the global human population is highly sensitized to a public health crisis of mass proportions.

## CONCLUSION

There is compelling evidence to suggest that women and girls in SSA carry a triple burden of vulnerability to NTDs, NCDs and poor reproductive health outcomes and that controlling for one may positively affect the outcomes for the other two. The current global health environment demands efficient, multidisciplinary, gender-sensitive and rights-based approaches to address the intersecting health, gender and economic issues facing women and girls throughout their life course. Hence, an integrative, multistakeholder workshop took up FGS, CC and CS as tracer disease-conditions, that impact woman’s health throughout their life course, to develop a framework for synergistic, sustainable and gender- and context-appropriate interventions to manage the NTD-NCD complex and additionally reproductive health. The overarching aim was 2-fold: to contextualize the interventional overlap of NCDs and NTDs for management, and scale-up this approach to include SRHR issues affecting girls and women in SSA; and to highlight how programmatic integration of all the key themes could enhance efficiencies of service delivery and better use of limited resources. Such an effort, while contributing to better health outcome in the region, also help accelerating the contribution to improving women’s health through the adoption of the SDG3, SDG5 and SDG10; on health and well-being, achieving gender equality, and reducing inequalities, respectively.

## Additional material


Online Supplementary Document
