# Supplementary material for: The challenges for women’s health in sub-Saharan Africa: Lessons learned from an integrative multistakeholder workshop in Gabon
Source: J Glob Health. 2021 Sep 4;11:02002. doi: 10.7189/jogh.11.02002 (PMC8442509; doi:10.7189/jogh.11.02002)
Supplement: Online Supplementary Document [file jogh-11-02002-s001.pdf]

Appendix S1: List of presenters / speakers

1. Prof. Dr. Ayola Akim Adegnika, CERMEL, GA
2. Dr. Maxime Agnandji, *CERMEL*, Gabon
3. Dr. Jonasse Solange Antimi, *Gabonese Ministry of Health*, GA
4. Dr. Amaya Bustinduy, *London School of Hygiene and Tropical Medicine*, UK
5. Prof. Dr. Clarissa Prazeres da Costa, *Technical University of Munich*, DE
6. Dr. Meral Esen, *University Clinic Tübingen*, DE
7. Dr. Robert Eya'mane, *Libreville Institute of Cancerology*, GA
8. Dr. Eveline Foguem, *Biyem-Assi District Hospital Yaoundé*, CM
9. Ms. Marrium Habib*, Technical University of Munich*, DE
10. Dr. Josiane Honkpehedji, *CERMEL*, GA
11. Dr. Laurianne James, *Libreville Institute of Cancerology*, GA
12. Prof. Dr. Stefanie Klug, *Technical University of Munich*, DE
13. PD Dr. Silvia Lobmaier, Klinikum Rechts-der-Isar, DE
14. Dr. Ulysse Minkobame, *Jeanne-Ebori Uni. Hospital Center for Mother & Child*, GA
15. Dr. Sibone Mocumbi, *Maputo Central Hospital*, MZ
16. Dr. Ghyslain Mombo-Ngoma, *CERMEL*, GA
17. Dr. Jutta Reinhard-Rupp, *Merck Global Health Institute*, CH
18. Dr. Andreas Ullrich, *Charité University Hospital Berlin*, *former WHO Geneva,* CH
19. Ms. Kathrin Vogg, Klinikum Rechts-der-Isar, DE
20. Dr. Rella Manego Zoleko, *CERMEL*, GA

CH: Switzerland
CM: Cameroon
DE: Germany
GA: Gabon
MZ: Mozambique
UK: United Kingdom

Appendix S2: Agenda of the workshop

Tuesday 21st January 2020	
8:30 -15:00	*Arrival and Registration of Participants*
15:00 -17:00	Opening Ceremony
	Speakers : Prof. Akim Adegnika, CERMEL, Gabon<br>Prof. Clarissa Prazeres da Costa, TUM, DE<br>Dr. Meral Esen, UKT, DE<br>Rep. Of Mayor of Lambaréné, Gabon
17:00 -19:00	Cocktail Reception
19:00	*Retire to Hotel*

Wednesday 22nd January 2020	
08:30 - 09:45	Plenary 1 : Female Genital Schistosomiasis
	Chairs: Prof. Peter Kremsner, CERMEL/ UKT, DE<br>Prof. Clarissa Prazeres da Costa, TUM, DE
08:30 - 09:15	Presenter: Dr. Amaya Bustinduy, LSHTM, UK<br>*« Female Genital Schistosomiasis, Schistosoma haematobium's neglected child»*
09:15 - 09:30	Presenters: Dr. G. Mombo-Ngoma, CERMEL, Gabon<br>*« Co-infection HIV and malaria during pregnancy: Interventions and Challenges »*
09:30 – 09:45	Presenter: Dr. R. Manego Zoleko, CERMEL, Gabon<br>*« Growth, nutritional status and mortality of cohort of children during their first year of life in rural and semi-urban areas of Gabon »*
09:45 - 10:30	Plenary 2 : Cervical Cancer
	Chairs: Dr. Meral Esen, UKT, DE
09:45 - 10:15	Presenter: Prof. Stefanie Klug, TUM, DE<br>*« Cervical Cancer and HPV in Sub Saharan Africa »*
10:15 - 10:30	Presenter: Dr. Andreas Ullrich, Charité Berlin, CH (online)<br>*« WHO's Global Policies against Cervical Cancer and National Implementation »*
10:30 - 10:45	Presenter: Dr. L. James, Institute of Cancerology, Gabon<br>*« Prognostic Factors for Cancer at the Institute of Cancerology in Gabon »*

10:45 -11 :00	*Coffee Break*	
11:00 - 12 :00	*Workshop 1: Female Genital Schistosomiasis* (Group 1)<br><br>Moderator: Dr. Jutta Reinhard-Rupp, Merck KGaA , CH	*Workshop 2: Cervical Cancer* (Group 2)<br><br>Moderator: Dr. Eveline Foguem, Biyem-Assi District Hospital, Cameroon
12:00 -13:00	*Workshop 1: Female Genital Schistosomiasis* (Group 2)<br><br>Moderator: Dr. Jutta Reinhard-Rupp, Merck KGaA, CH	*Workshop 2*: Cervical Cancer (Group 1)<br><br>Moderator: Dr. Eveline Foguem, Biyem-Assi District Hospital, Cameroon
13:00 - 14:30	*Lunch*	
14:30 - 16:30	Round table on FGS and Cervical Cancer	
	Chair: Dr. Sibone Mocumbi, Hôpital Central de Maputo, Mozambique<br><br>Experts: Dr. L. James, Institute of Cancerology, Gabon<br>Dr. Amaya Bustinduy, LSHTM, UK<br>Prof. Stefanie Klug, Technical University of Munich, DE<br>Dr. Jutta Reinhard-Rupp, Merck KGaA, CH	
16:30 - 17:30	*Coffee Break & Networking*	
16:30 – 17:30	Summary and Presentation of Workshop Outcomes<br><br>Presenters: Dr. Eveline Foguem, Biyem-Assi District Hospital, Cameroon<br>Dr. Jutta Reinhard-Rupp, Merck KGaA, CH	
19:00 - 21:00	*Dinner*	

	Thursday 23rd January 2020
08:30 - 09:35	Plenary 3 : Cesarean Section
	Chairs: Dr. Sibone Mocumbi, Hôpital Central de Maputo, Mozambique
08:30 - 09:15	Presenters: Dr. Silvia Lobmaier, Klinikum Rechts-der-Isar Munich, TUM, DE<br>*« Problems of Cesarean section in low-income countries »*
09:15 - 09:30	Dr. Robert Eya'mane, Libreville Institute of Cancerology, Gabon<br>*« SCREENING OF CERVICAL CANCER IN GABON BY VISUAL INSPECTION AFTER APPLICATION OF ACETIC ACID AND LUGOL »*
09:30 – 10:15	Plenary 4 : Women's Health in Gabon
	Chairs: Prof. Ayôla Akim Adegnika, CERMEL, Gabon<br>Dr. Eveline Foguem, Biyem-Assi District Hospital, Cameroon
09:30 - 09:45	Presenters: Dr. Josiane Honkpehedji, CERMEL, Gabon

	« Maternal Polyparasitism is Associated with Low Birth Weight in Gabon »
09:45 - 10:00	Presenter: Dr. Ulysse Minkobame, Jeanne-Ebori Uni. Hospital Center MCH, Gabon<br>*« The benefit of Robson's classification in the practice of cesarean section in 2018 in Libreville »*
10:00 – 10:15	Presenter : Dr. Jonasse Solange Antimi, Gabonese Ministry of Health, GA<br>*« Maternal and Child Health in Gabon »*
10:15 – 10:30	*Coffee Break*
10:30 – 12:30	Round Table on Cesarean Section
	Chair: Dr. Maxime Agnandji, CERMEL, Gabon
	Participants: Dr. Silvia Lobmaier, Klinikum Rechts-der-Isar Munich, TUM, DE<br>Dr. Ulysse Minkobame, Jeanne-Ebori Uni. Hospital Center MCH, Gabon<br>Dr. Eveline Foguem, Biyem-Assi District Hospital, Cameroon
12:00 - 13:30	*Lunch*
13:30 -15:30	Hands-On Teaching Modules<br>*« Management of problems during and after birth »*<br>*« Low-cost cervical inspection for diagnosis of FGS/CC »*
	Instructors: Dr. Silvia Lobmaier, Klinikum Rechts-der-Isar Munich, TUM, DE<br>Ms. Kathrin Vogg, Klinikum Rechts-der-Isar Munich, TUM, DE<br>Dr. Amaya Bustinduy, LSHTM, UK
15:45 - 16:45	Summary and Presentation on Outcomes of the Round Table and the Teaching Modules
16:45 - 17:45	Panel Discussion<br>*« Framework of future activities based on the messages to be retained from the workshops »*
17:45 - 18:00	*Coffee Break*
18:00 – 18:30	Presentation of Concept and Main Messages of Workshop White Paper<br>Speaker: Marrium Habib, TUM, DE
18:30 – 19:00	Closing Ceremony<br>Speakers: HelmVit & CERMEL teams
19:00 - 21:00	*Farewell Dinner*
